# Erythrodermic psoriasis with early diagnostic uncertainty requiring multidisciplinary rheumatological management: A case report

**DOI:** 10.3892/mi.2026.330

**Published:** 2026-07-10

**Authors:** Shilpa Raman Danagara, Tarun Selvarajan, Lavanya Kannekanti, Aleeza Qamar, Ashley Flowers, Sarwat Umer

**Affiliations:** 1 Department of Internal Medicine, LSU Health Shreveport, Shreveport, LA 71103, USA; 2 Center of Excellence for Arthritis and Rheumatology, LSU Health Shreveport, Shreveport, LA 71103, USA; 3 Department of Pathology, LSU Health Shreveport, Shreveport, LA 71103, USA

**Keywords:** erythrodermic psoriasis, toxic epidermal necrolysis, Stevens-Johnson syndrome, diagnostic dilemma, intravenous immunoglobulin, IL-17 inhibitors, secukinumab, multidisciplinary management

## Abstract

Erythrodermic psoriasis is a rare and potentially life-threatening variant of psoriasis characterized by diffuse erythema and scaling involving the majority of body surface area. The clinical presentation may overlap with severe cutaneous adverse reactions, such as Stevens-Johnson syndrome and toxic epidermal necrolysis (TEN), creating diagnostic uncertainty and therapeutic challenges. Standard treatment includes cyclosporine or biological therapy, whereas intravenous immunoglobulin (IVIG) is traditionally reserved for TEN. The present case report describes the case of a 42-year-old woman with biopsy-proven psoriasis and polycythemia vera who presented with rapidly progressive erythroderma involving ~90% of her body surface area following cyclosporine dose escalation. Marked systemic inflammation and TEN-like features prompted treatment with IVIG at 400 mg/kg/day for 5 consecutive days, resulting in a marked clinical improvement. She was subsequently transitioned to secukinumab for maintenance therapy. The present case report highlights IVIG as a potential rescue therapy in severe erythrodermic psoriasis when diagnostic overlap with TEN exists and rapid disease control is required.

## Introduction

Psoriasis is a common chronic immune-mediated inflammatory disease. Although plaque psoriasis represents the most common phenotype, erythrodermic psoriasis (EP) is an uncommon variant associated with substantial morbidity and potential mortality. EP is characterized by generalized erythema, scaling, exfoliation and systemic manifestations that arise from extensive cutaneous inflammation and impaired barrier function. The widespread involvement of skin may lead to fluid imbalance, thermoregulatory dysfunction, electrolyte abnormalities and high-output cardiac stress (1).

EP may develop in patients with longstanding plaque psoriasis or arise abruptly in the setting of medication changes, systemic corticosteroid withdrawal, infection, or poorly controlled disease (1). Clinically, erythrodermic psoriasis may closely resemble severe drug eruptions, particularly Stevens-Johnson syndrome and toxic epidermal necrolysis (TEN). Differentiation between these entities is critical because management strategies differ significantly. TEN is frequently treated with intravenous immunoglobulin (IVIG) or cyclosporine, whereas erythrodermic psoriasis is typically managed with rapid-acting systemic immunosuppression such as cyclosporine, methotrexate, or biologic agents targeting tumor necrosis factor (TNF), interleukin (IL)-17, or IL-23 pathways (1,2).

Although IVIG has an established role in TEN, it is not used as standard therapy for EP; however, some previous studies have demonstrated its potential benefit in rescue settings (1,3). The present case report describes a severe case of EP that presented with early diagnostic uncertainty regarding toxic epidermal necrolysis and improved following IVIG, emphasizing the diagnostic complexity and therapeutic considerations from a multidisciplinary rheumatological and dermatological perspective.

## Case report

A 42-year-old woman with a history of biopsy-confirmed psoriasis diagnosed in July, 2025, polycythemia vera (previously treated with hydroxyurea, but discontinued due to adverse effects), essential hypertension, hyperlipidemia and prior non–ST-elevation myocardial infarction presented to Ochsner LSU Health Shreveport-Academic Medical Center (Shreveport, LA, USA), with a worsening generalized rash over a period of 2 weeks. Her psoriasis had initially been managed by dermatology with cyclosporine (Sandimmune) 100 mg/day (~1.5 mg/kg/day), intentionally commenced at the lower end of the dosing range in July, 2025, together with topical corticosteroids. At a follow-up visit in October, disease activity had modestly improved, but remained active, and therapy was escalated to cyclosporine modified (Neoral) at 150 mg/day (~2.3 mg/kg/day at a body weight of 66 kg), with a planned overlap and transition to guselkumab once insurance authorization was obtained. A tumor necrosis factor inhibitor (adalimumab) had been considered previously, but was never initiated and was regarded as contraindicated given her active polycythemia vera; the planned biological bridge was therefore the IL-23, guselkumab, for which authorization was pending.

During a telemedicine visit in October, the escalation of cyclosporine dosing was recommended due to persistent disease activity. Shortly after this adjustment, she developed rapidly progressive diffuse erythema, severe burning pain, scaling and swelling, impairing her ability to perform activities of daily living. She described intense burning and blister-like discomfort, but denied fevers, dyspnea, new medications, or systemic infectious symptoms. Although a urine toxicology screen from an outside facility was positive for cocaine, the patient denied illicit drug use.

**Figure 1:**
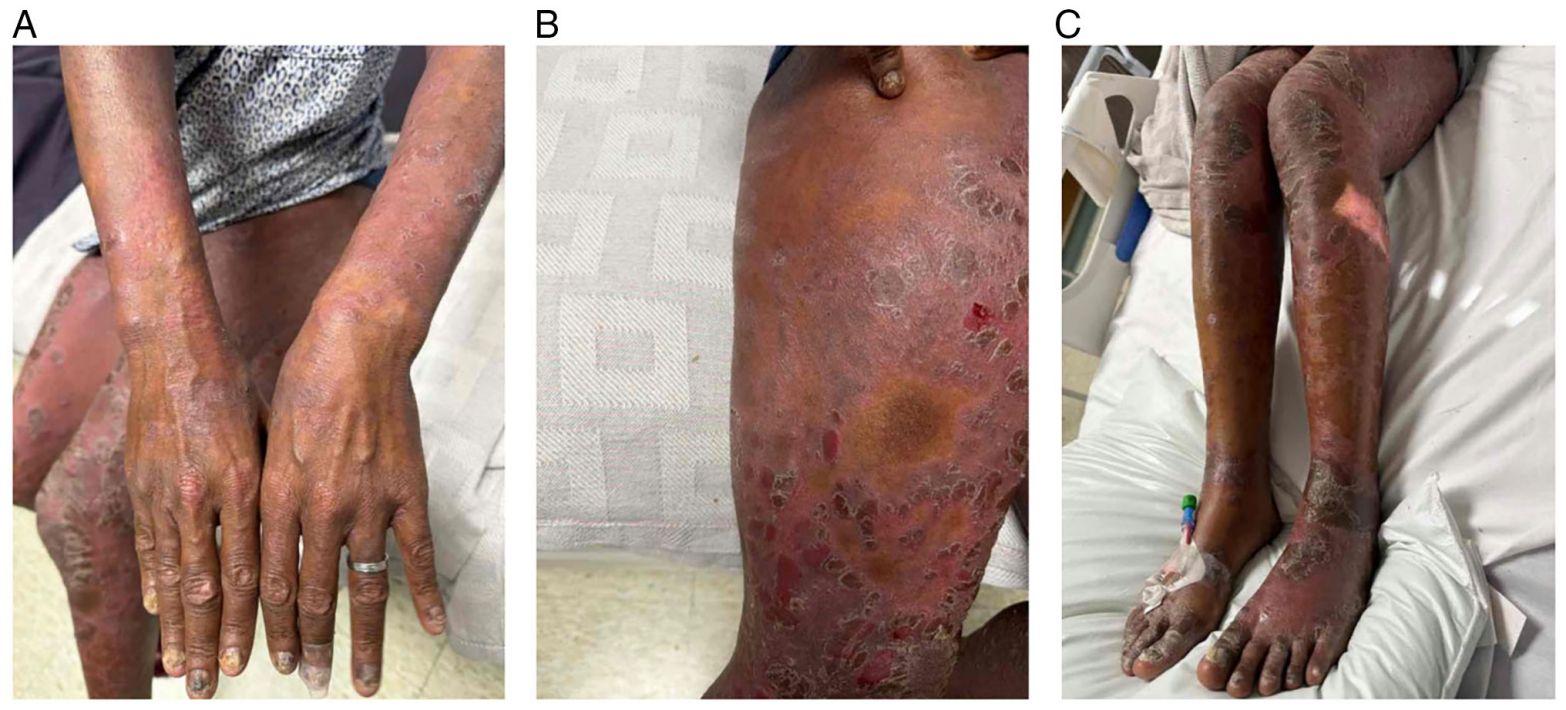
Clinical presentation of the patient with erythrodermic psoriasis. (A) Diffuse erythema with overlying scaling involving the upper extremities and hands, demonstrating widespread inflammatory involvement with desquamation. (B) Extensive erythematous plaques with superimposed scaling and areas of erosion over the trunk, highlighting the severity of cutaneous involvement. (C) Bilateral lower extremity involvement with diffuse erythema and hyperkeratosis with areas of diffuse scaling and desquamation consistent with erythrodermic psoriasis, along with focal crusting secondary to inflammation.

Upon admission to Ochsner LSU Health Shreveport- Academic Medical Center on November 9, 2025, her blood pressure was 155/93 mmHg, pulse 87 beats per minute, respiratory rate 20 breaths per minute, and temperature 98.8˚F (37.1˚C). Although she appeared uncomfortable, she was hemodynamically stable. A dermatological examination revealed diffuse erythematous scaly plaques involving ~90% of her body surface area, including the circumferential involvement of bilateral upper and lower extremities, anterior chest, abdomen and back (Fig. 1). Fine scaling was noted in intertriginous areas. The lips demonstrated mild scaling without mucosal erosions. The Nikolsky sign was positive, with focal inducible epidermal shearing on lateral pressure, and superficial desquamation was present diffusely. However, there was no spontaneous full-thickness sheet-like epidermal detachment, no bullae, and no areas of frank necrosis. Palms and soles were spared. A musculoskeletal examination revealed no synovitis or joint effusions. Cardiopulmonary and abdominal examinations did not reveal any notable findings.

A laboratory evaluation demonstrated leukocytosis with a white blood cell count of 18.27 K/µl, a hemoglobin level of 10.3 g/dl with microcytosis (mean corpuscular volume, 68 fl) and thrombocytosis ranging from 725 to 808 K/µl. The level of C-reactive protein (CRP) was markedly elevated at 100 mg/l and the erythrocyte sedimentation rate (ESR) was 26 mm/h. Antinuclear antibody and desmoglein antibodies were negative, and immunoglobulin levels did not exhibit any notable abnormality (IgG, 1,265 mg/dl; IgM, 50 mg/dl; IgE, 79 IU/ml), with a mildly elevated IgA (355 mg/dl). These findings reflected significant systemic inflammation and known hematological abnormalities associated with polycythemia vera and iron deficiency.

**Figure 2:**
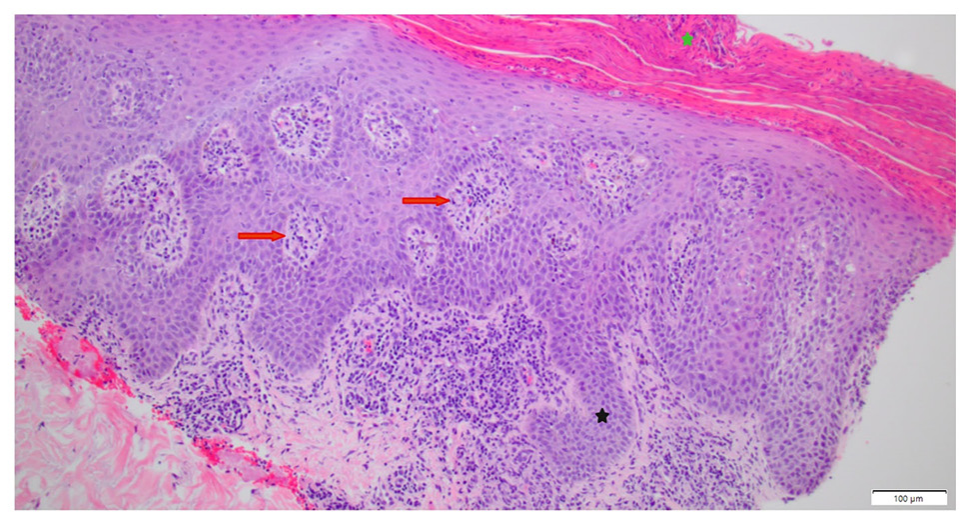
Histopathological findings of erythrodermic psoriasis. Punch biopsy demonstrates diffuse hyperparakeratosis with attenuation of the granular layer (green asterisk). Collections of neutrophils are present within the stratum corneum, consistent with Munro microabscesses (red arrows). There is marked acanthosis with elongation and bridging of rete ridges (black asterisk), along with lymphocytic exocytosis and mild spongiosis. No well-formed spongiform pustules of Kogoj are identified. Dilated capillaries are observed within the dermal papillae. Periodic acid-Schiff staining is negative for fungal organisms. No epidermal necrosis is identified. Original magnification, x10; scale bar, 100 µm.

A punch biopsy of the involved skin and a histopathological analysis were performed. Skin sections were formalin-fixed (9 h at 25˚C), paraffin-embedded, and cut at a thickness of 4 µm. Hematoxylin and eosin staining (Leica Biosystems) was performed at 70˚C for 1 h and 10 min, and the sections were examined under a light microscope (Leica Microsystems GmbH). This analysis of the skin samples demonstrated diffuse hyperparakeratosis, attenuation of the granular layer, clusters of neutrophils within the stratum corneum, marked acanthosis with elongation and bridging of rete ridges, spongiosis with lymphocyte exocytosis, and dilated capillaries within dermal papillae. Notably, there was no evidence of full-thickness epidermal necrosis (Fig. 2). These histopathological findings supported a diagnosis of psoriasis rather than TEN.

The patient was admitted for multidisciplinary management involving dermatology, rheumatology, hematology, allergy/immunology and burn surgery services. High-potency topical corticosteroids were initiated, including triamcinolone 0.1% twice daily and later, betamethasone dipropionate 0.05%. Supportive care focused on fluid balance, electrolyte monitoring and temperature regulation. Given her underlying polycythemia vera and thrombocytosis, prophylactic anti-coagulation with enoxaparin at 40 mg daily was administered. Pain was managed with intravenous morphine every 8 h as needed for 2 days, followed by oral oxycodone 5 mg every 6 h, as needed. Systemic corticosteroids were intentionally avoided due to the well-recognized risk of rebound psoriasis flares following taper.

Dermatology considered resuming cyclosporine at 3 mg/kg/day divided twice daily, with a maximum of 100 mg twice daily. However, given the rapid progression despite cyclosporine therapy, elevated levels of inflammatory markers (CRP, 100 mg/l; ESR, 26 mm/h), and persistent diagnostic concern for TEN, the Rheumatology Department recommended the initiation of IVIG at 400 mg/kg/day for 5 consecutive days (total dose 2 g/kg), commencing on November 11, 2025.

Within 48 h of IVIG initiation, the patient reported a marked reduction in the sensation of burning pain. By the completion of the 5-day course on November 15, the erythema and scaling had substantially improved, and she regained functional mobility. No thrombotic or infusion-related complications occurred despite her prothrombotic risk profile. Following clinical stabilization, she was transitioned to secukinumab for long-term disease control, provided through sample loading doses prior to discharge. She was discharged home in stable condition with close outpatient follow-up arranged with the Dermatology, Rheumatology and Hematology Departments.

**Figure 3:**
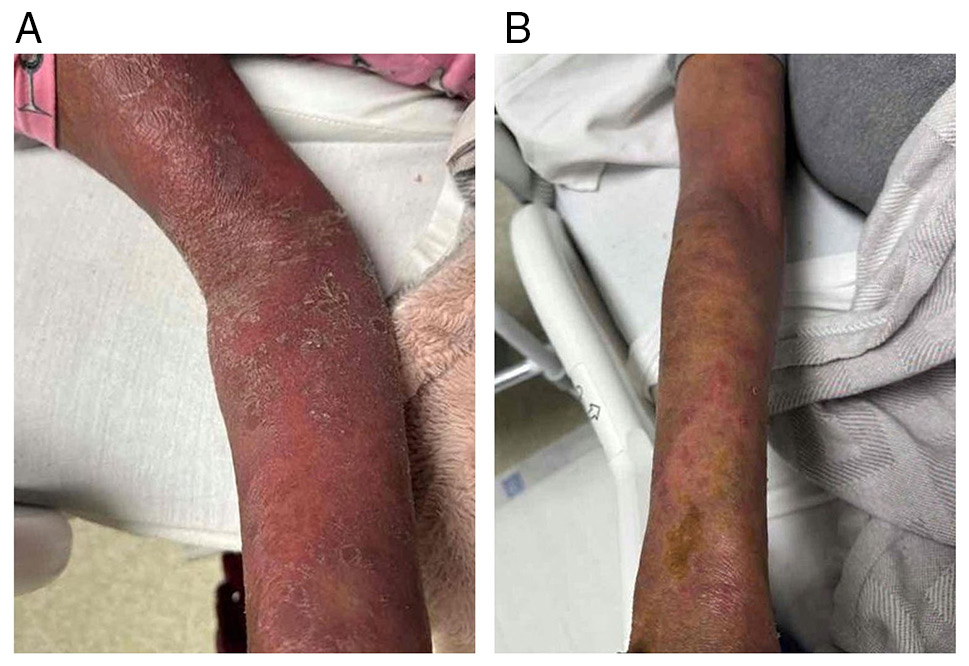
Clinical response of the patient to IVIG. (A) Right forearm at presentation, demonstrating confluent erythema with fine scaling consistent with erythrodermic psoriasis. (B) Right forearm following IVIG, with the resolution of erythema and scaling and residual post-inflammatory hyperpigmentation. IVIG, intravenous immunoglobulin.

The clinical response of the patient was supported by objective and semi-objective measures. The white blood cell count decreased from a peak of 18.27 K/µl on November 9 toward the near-normal range at 12.68 K/µl by the completion of IVIG on November 15, paralleling clinical improvement, with expected day-to-day variation; the platelet count increased over the same interval, consistent with her known polycythemia vera and not interpreted as a marker of treatment response. A repeat C-reactive protein was not obtained during the admission, which the authors acknowledge as a limitation. Body surface area involvement progressed from ~20% at initial presentation to ~90% at admission, with the subsequent resolution of erythema and scaling. At presentation, severe burning pain rendered the patient unable to perform activities of daily living; this improved substantially following IVIG, with restoration of functional mobility and self-care. Representative images of the right forearm before and after IVIG are presented in Fig. 3. The overall clinical course of the patient is summarized in Table I.

## Discussion

EP represents one of the most severe inflammatory forms of psoriasis, characterized by diffuse erythema and scaling involving the majority of the body surface area. The pathophysiology of EP involves extensive immune dysregulation, with the overactivation of the Th17 and Th1 pathways, leading to the increased expression of cytokines, such as IL-17, IL-23 and TNF-α. This cascade promotes keratinocyte hyperproliferation and widespread inflammation that manifests clinically as confluent erythema with systemic complications (1,3-5). Distinguishing EP from severe cutaneous adverse reactions, such as Stevens-Johnson syndrome or TEN is crucial, as the therapeutic approaches and prognoses of the two conditions differ significantly (1,2). In the case described herein, the diagnostic uncertainty inherent in overlapping presentations, combined with the atypical clinical course and severe functional impairment of the patient, led clinicians to consider IVIG traditionally a therapy for TEN as a rescue intervention (1,2).

Notably, a positive Nikolsky sign was documented by multiple services and superficial desquamation was present, heightening initial concern for TEN and contributing to the diagnostic uncertainty that informed the decision to administer IVIG. However, a histopathological analysis demonstrated changes characteristic of psoriasis without full-thickness epidermal necrosis and the clinical course, including the absence of a culprit drug, the background of biopsy-proven psoriasis and the response to therapy, ultimately supported the diagnosis of EP. The present case report underscores that the Nikolsky sign, while suggestive, is not by itself sufficient to distinguish EP from epidermal necrolysis. A comparison of the distinguishing features is summarized in Table II.

The selection of IVIG over other rapid-acting options also warrants an explanation. IVIG was selected as a diagnosis-agnostic bridge to rapid control. Further cyclosporine escalation provided limited confidence, as the patient was already progressing on a recently escalated dose; the effects of methotrexate and acitretin are too gradual for a 90% body surface area emergency; and a biologic, although planned for maintenance, would not be expected to achieve control within 48 h. TNF inhibitors were contraindicated given her active polycythemia vera. Persistent diagnostic overlap with TEN, reinforced by a positive Nikolsky sign and superficial desquamation, meant that IVIG addressed both leading possibilities simultaneously, and it was therefore administered for both suspected TEN and severe erythrodermic psoriasis. Rapid control was achieved despite her prothrombotic profile, with enoxaparin prophylaxis and without thrombotic complication.

Beyond TEN, other causes of erythroderma were considered. Drug-induced erythroderma was possible; although a urine toxicology screen was positive for cocaine, the patient denied illicit drug use, no new culprit medication was identified, and the histopathological analysis and background of biopsy-proven psoriasis argued against a primary drug eruption. Drug reaction with eosinophilia and systemic symptoms was considered less likely, given the absence of peripheral eosinophilia, facial edema, lymphadenopathy and internal organ involvement, and an incompatible temporal course. Sézary syndrome was specifically considered given its importance in the evaluation of erythroderma; there was no lymphadenopathy on examination, and skin histopathology demonstrated psoriasiform changes without atypical epidermotropic lymphocytes. A peripheral blood smear and flow cytometry were not performed, which the authors acknowledge as a limitation; the rapid therapeutic response and overall clinicopathologic picture nonetheless favored EP.

Over the past decade, biological therapies have transformed the management of psoriasis by targeting specific cytokines implicated in disease pathogenesis. Traditional systemic therapies, such as methotrexate or cyclosporine are often used initially in acute EP due to their relatively rapid onset of action (1,6). However, the development of biologics aimed at IL-17 and IL-23 pathways has introduced highly effective, targeted options that can achieve profound and sustained cutaneous clearance (7-9). These agents have been particularly advantageous in EP due to their ability to interrupt inflammatory networks directly responsible for widespread disease activity.

Recent systematic analyses focusing on biological treatment outcomes in EP provide valuable insights into real-world efficacy. A 2025 systematic review (7) reported pooled PASI 75 response rates of ~70% at 24 weeks, with superior short-term efficacy for IL-17 inhibitors compared with IL-23 and TNF-α inhibitors; a broader systematic review of biologics in erythrodermic psoriasis has reported comparable findings (9). Across extended follow-up (28-148 weeks), IL-17 biologics maintained high response rates, while IL-23 inhibitors demonstrated substantial efficacy with favorable durability (7). These findings reinforce IL-17 blockers as particularly potent options for rapid disease control, while IL-23 inhibitors remain effective and show promise for longer-term maintenance therapy.

Within the IL-17 class, agents, such as secukinumab, ixekizumab and brodalumab have been consistently associated with high levels of skin clearance in EP and plaque psoriasis (8,10). Real-world, single-center data likewise support the efficacy and tolerability of secukinumab, with notable reductions in erythema and scaling observed in clinical practice (10). For patients with severe disease activity and a high inflammatory burden, IL-17 inhibition may therefore be preferred when a rapid clinical response is required. However, clinicians should balance this with individual risk profiles, as IL-17 inhibitors have been associated with an increased risk of mucocutaneous candidiasis and injection site reactions, although their overall safety profile remains favorable (8,10).

IL-23p19 inhibitors, including guselkumab and risankizumab, have demonstrated potent efficacy in psoriasis and are increasingly reported in EP (9,11). Emerging case reports and clinical experience, including the successful treatment of EP with guselkumab, suggest that these agents can achieve sustained disease control with favorable safety profiles (11,12). Additionally, registry data indicate that biologics overall exhibit good drug survival and long-term tolerability in psoriasis management (13). Though EP-specific data for IL-23 inhibitors are more limited, the favorable long-term drug survival and safety profiles observed in chronic plaque psoriasis support their use as maintenance options following initial clearance.

Despite these encouraging results, large randomized controlled trials specifically focusing on EP are lacking, largely due to its rarity. The majority of the available evidence is derived from retrospective cohorts, case series and meta-analysis of pooled data, highlighting the need for dedicated prospective studies (7). Nevertheless, current evidence supports the efficacy and tolerability of biologics in EP, with IL-17 inhibitors providing rapid disease control and IL-23 inhibitors providing durable long-term management.

In the present case report, the decision to transition to secukinumab at discharge was supported by evidence demonstrating the strong efficacy of IL-17 inhibitors in EP (8,10). However, initial rapid disease control was achieved with IVIG, suggesting a potential role for this therapy as a rescue option in cases where conventional systemic agents are insufficient, contraindicated, or when diagnostic overlap with TEN complicates early treatment decisions (1,2). The present case report emphasizes the importance of flexible therapeutic strategies and the need for individualized treatment plans based on disease severity, comorbidity profiles and emerging evidence.

However, the present case report has several limitations, which should be mentioned. As it reports a single case, causality cannot be established. The temporal improvement following IVIG is suggestive, but confounded by concurrent cyclosporine activity, intensive supportive inpatient care and the natural history of the disease; a contributory rather than singular role for IVIG cannot be excluded. Therefore, IVIG can be used as a potential rescue option warranting further study, rather than a proven cause of resolution.

In conclusion, the present case report highlights the diagnostic and therapeutic complexity of EP, particularly when early diagnostic uncertainty with TEN arises from discordant, overlapping features. In severe, refractory presentations requiring rapid disease control, IVIG may represent a viable rescue therapy. Multidisciplinary collaboration between the Dermatology and Rheumatology Departments was essential in establishing the diagnosis and guiding management. Further prospective studies are warranted to clarify the role of IVIG in EP and to determine which patients may benefit most from this approach.

## Figures and Tables

**Table I:** Timeline of the clinical course, treatment and response of the patient.

Date	Event	Skin/clinical findings	Treatment
July, 2025	Psoriasis diagnosed (biopsy)	Widespread plaques including hands/feet; nail dystrophy; BSA ~20%	Topicals only, ineffective
July 31, 2025	Initial dermatology visit	Severe; BSA ~20%	Cyclosporine (Sandimmune) 100 mg/day (~1.5 mg/kg/day); guselkumab planned pending TB testing
October, 2025	Dermatology follow-up	Improved, but still active	Cyclosporine escalated to Neoral 150 mg/day (~2.3 mg/kg/day); guselkumab prescribed, authorization pending
Early November, 2025	Rapid progression	Diffuse erythema, severe burning pain, desquamation; BSA ~90%; positive Nikolsky sign	-
November 9, 2025	Admission	~90% BSA; unable to perform ADLs	Topicals, supportive care, enoxaparin; systemic steroids avoided
November 9, 2025	Peak inflammation	-	WBC, 18.27; CRP, 100; ESR, 26; platelets, 725-808
November 11, 2025	IVIG initiated	~90% BSA	IVIG 400 mg/kg/day for 5 days (2 g/kg)
November 13, 2025	Early response (+48 h)	Reduced burning pain	-
November, 15 2025	IVIG completed	Erythema/scaling improved; mobility regained; WBC 12.68	-
November 18, 2025	Discharge	Stabilized; resolving erythema	Secukinumab loading; outpatient follow-up

ADLs, activities of daily living; BSA, body surface area; CRP, C-reactive protein (mg/l); ESR, erythrocyte sedimentation rate (mm/h); IVIG, intravenous immunoglobulin; TB, tuberculosis; WBC, white blood cell count (K/µl).

**Table II:** Distinguishing features of erythrodermic psoriasis versus Stevens-Johnson syndrome/toxic epidermal necrolysis in the patient in the present case report.

Feature	The patient in the present case report	Classic SJS/TEN
Background	Biopsy-proven psoriasis	Usually absent
Nikolsky sign	Positive (documented by two services)	Positive
Desquamation	Superficial desquamation present	Sheet-like full-thickness sloughing
Epidermal detachment	No spontaneous full-thickness detachment	Hallmark (SJS, <10% TEN >30% BSA)
Targetoid lesions	Absent	Characteristic
Mucosal involvement	Mild lip scaling; no erosions	Erosive, ≥2 sites typical
Skin pain	Severe burning, limiting ADLs	Prominent
Culprit drug	No new culprit drug	New drug typically 1-4 weeks prior
Histopathology	Parakeratosis, neutrophils, acanthosis; no full-thickness necrosis	Full-thickness necrosis, basal apoptosis
SCORTEN	Not meaningfully applicable (no detachment)	Validated prognostic score

ADLs, activities of daily living; BSA, body surface area; SCORTEN, severity-of-illness score for toxic epidermal necrolysis; SJS, Stevens-Johnson syndrome; TEN, toxic epidermal necrolysis.

## Data Availability

The data generated in the present study may be requested from the corresponding author.
